# Triggers and Effectors of Oxidative Stress at Blood-Brain Barrier Level: Relevance for Brain Ageing and Neurodegeneration

**DOI:** 10.1155/2013/297512

**Published:** 2013-03-07

**Authors:** Ana-Maria Enciu, Mihaela Gherghiceanu, Bogdan O. Popescu

**Affiliations:** ^1^Laboratory of Molecular Medicine, “Victor Babeş” National Institute of Pathology, 99-101 Splaiul Independenţei, 050096 Bucharest, Romania; ^2^Department of Cellular and Molecular Medicine, School of Medicine, “Carol Davila” University of Medicine and Pharmacy, 8 Eroilor Sanitari, 050474 Bucharest, Romania; ^3^Department of Neurology, Colentina Clinical Hospital (CDPC), School of Medicine, “Carol Davila” University of Medicine and Pharmacy, 19-21 Sos. Stefan cel Mare, 020125 Bucharest, Romania

## Abstract

As fundamental research advances, it is becoming increasingly clear that a clinically expressed disease implies a mixture of intertwining molecular disturbances. Oxidative stress is one of such pathogenic pathways involved in virtually all central nervous system pathologies, infectious, inflammatory, or degenerative in nature. Since brain homeostasis largely depends on integrity of blood-brain barrier (BBB), many studies focused lately on BBB alteration in a wide spectrum of brain diseases. The proper two-way molecular transfer through BBB depends on several factors, including the functional status of its tight junction (TJ) complexes of proteins sealing neighbour endothelial cells. Although there is abundant experimental work showing that oxidative stress associates BBB permeability alteration, less is known about its implications, at molecular level, in TJ protein expression or TJ-related cell signalling. In this paper, oxidative stress is presented as a common pathway for different brain pathogenic mechanisms which lead to BBB dysregulation. We revise here oxidative-induced molecular mechanisms of BBB disruption and TJ protein expression alteration, in relation to ageing and neurodegeneration.

## 1. Introduction

It has been extensively proven that a large array of neurological diseases and brain ageing itself are associated with oxidative stress [1–3]. Multiple sclerosis, stroke, brain tumours, and neuroinfections are conditions which associate both reactive oxygen species (ROS) aggression and blood brain barrier (BBB) impairment as well-proven pathogenic mechanisms. Relatively recent data documents BBB disruption not only in vascular or inflammatory brain diseases but in neurodegenerative disorders as well, where oxidative stress plays an important role in the pathogenic scenario [4, 5]. Whether oxidative damage is an important and early event in BBB alteration process, it is not established so far.

**Figure 1:**
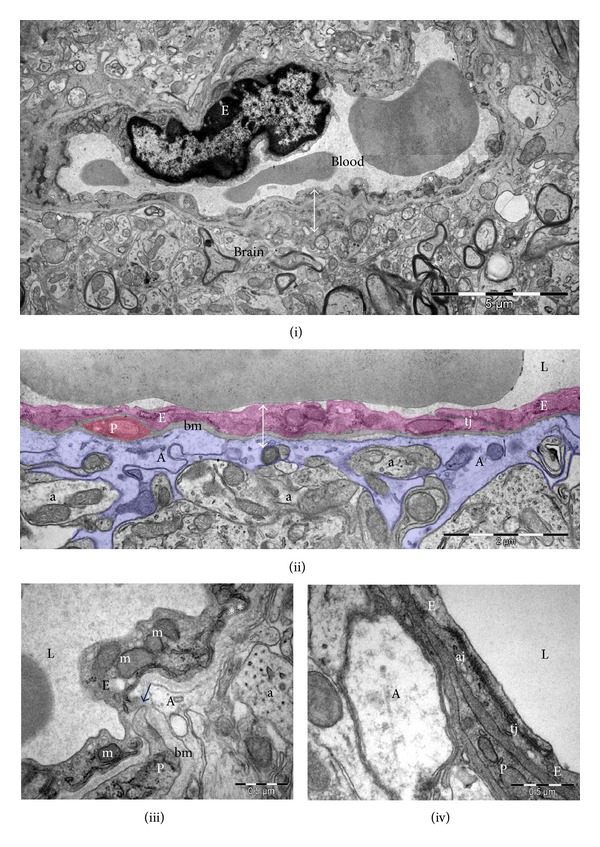
Ultrastructure of blood-brain barrier (↔). (i) Overall electron microscopy image of a cerebral capillary. (ii) Blood-brain barrier components: endothelial cells (E, purple coloured), pericytes (P, brown coloured), basement membrane (bm), and end-feet of astrocytes (A, blue coloured). (iii) Cerebral capillaries have nonfenestrated endothelial cells with numerous mitochondria (m) and rare pinocytotic vesicles (*). Direct membrane-membrane contacts (arrow) often occur between endothelial cells and pericytes. (iv) Tight (tj) and adherens (aj) junctions seal the continuous capillary endothelium. Cerebral capillary lumina (L), axons (a).

BBB is the interface between the periphery of circulatory system and central nervous system. The endothelial cells are the primary components of the BBB, responsible for the controlled environment of the brain. These cells lack fenestrations and have increased mitochondrial content, minimal pinocytotic activity, and a low number of caveolae. A 30–40 nm thin basement membrane is found between endothelial and neighbouring glial cells [6, 7]. At the BBB level, paracellular transport is restricted by tight junctions (TJs), allowing a peculiar “sealing” capacity. However, other cell types—pericytes, astrocytes, and neurons—are required for an accurate organization and function of BBB, not necessarily through direct contact with endothelial cells (Figure 1). *Pericytes* are the only cell type to intimately connect with endothelial cells, as they lay embedded within the endothelial basement membrane—a fibrillary structure of collagen IV, laminins, and proteoglycans. Pericytes strengthen the barrier integrity and their loss opens the BBB in an age-dependent manner [8]. Recently, pericytes have been added to the classical *in vitro* 2-cell type model of BBB (coculture of endothelial and astrocytes) [9]. *Astrocytes* are separated from endothelial cells by the basement membrane around which they extend cell processes called end-feet. Hence, no cell-to-cell junctions are involved in this case, but the molecular flow of information between the two cell types is vital for BBB embryonic development [10] and adult life BBB integrity [11]. *In vitro* studies indicate astrocytes as regulators of TJ tightness and polarized distribution of transporters at endothelial level [12]. Furthermore, coculture of astrocytes with epithelial (other than brain endothelial) cells leads to induction of BBB properties [13, 14] and this is now a common practice in *in vitro* BBB models. *Neurons* are not morphologically involved in BBB formation, but numerous myelinated and nonmyelinated axons are found in close proximity of brain capillaries. The current model of brain homeostasis is based on the neurovascular unit, comprising cellular elements of BBB along with the neurons to which they connect into a functional network [15].

The BBB is functionally characterized by highly restrictive transbarrier transport, due to sealing of paracellular pathway by TJs and low transcytotic traffic through caveolae [10]. Transport of virtually all nondiffusible, nonlipidic molecules is controlled through specific carriers present on both sides of endothelial cells, in a time- and concentration-dependent manner. Consequently, quantification of large protein (albumin, dextran) traffic from blood to nervous tissue is an indicator of “tightness” of cell-to-cell endothelial junctions. A barrier “tightness” or “leakiness” is given by expression and molecular organization of different TJ species, which in the case of BBB are unique. In fact, clusters of densely packed molecules form the interendothelial junctions that contain specific components for both adherens and tight junctions. 

The TJ is an intricate macromolecular complex [6, 16] formed by:

- integral membrane proteins: claudins (claudin-1, 2, 3, 5, 11, 12, 18), MARVEL (the myelin and lymphocyte protein (MAL) and related proteins for vesicle trafficking and membrane link) proteins (occludin, tricellulin/marvelD2 and marvelD3), junctional adhesion molecules (JAMs), endothelial cell selective adhesion molecule (ESAM), and so forth;
- cytoplasmic proteins: zonula occludens proteins (ZO-1, 2, 3), afadin (AF-6), calcium/calmodulin dependent serine protein kinase (CASK/LIN-2) from membrane-associated guanylate kinase proteins (MAGUK family), actin binding protein (cingulin), small G-proteins (Rho, Rac, Cdc42), ZO-1-associated nucleic acid binding protein (ZONAB), cyclin-dependent kinase-4 (CDK-4), and so forth;
- actin cytoskeleton.

For a long while, the TJ complexes were considered static structures but new data support a dynamic model of barriers and also suggest that regulation of TJ openings and closings may provide sensitive means to modulate barrier function without changing protein expression [17]. 

*In vivo* and *in vitro* molecular studies of TJ proteins show that alteration of BBB in neurodegeneration usually cooccurs with modified TJ protein expression. As already mentioned, molecular organization of TJs is responsible for the “leakiness” of the BBB, which physiologically is more tight than other epithelial sites, a fact illustrated by a transepithelial resistance 50 times higher than other epithelia [18]. This peculiarity is not only a consequence of protein composition, but also of cellular sensitivity to microenvironment [19].

Although deleterious effects of oxidative stress on neuronal and glial populations in healthy aged and dementia brains are well stated, less is known about its consequences on endothelial cells, BBB, and tight junction protein expression. Based on the functional concept of neurovascular units, the presumption of BBB alteration in a neuronal/glial oxidative stress microenvironment is a plausible theory, possibly involving oxidative stress-related molecules. Furthermore, age is a certain inductor of BBB alteration, as briefly discussed in the following section; therefore, occurrence of oxidative stress in early stages of neurodegeneration might initiate tight junction impairment.

However, *in vitro* experimental setups used to decipher TJ protein alterations in oxidative environment are very variable in terms of culturing conditions. Most authors acknowledged the need to replicate the results by glial-endothelial cocultures, use of conditioned media, or *in vivo* conditions [20, 21]. Thus, the results are sometimes conflicting or even contradictory (see Table 1). 

## 2. Endothelial Ageing and Tight Junctions in Aged Blood-Brain Barrier

Ageing is an independent factor associated with endothelial dysfunction even in the absence of other cardiovascular risk factors [22]. Aged endothelium showed a defective response to certain vasodilators [23], related to reduced NO-mediated dilatation [24], oxidative stress, and vascular inflammation [25]. Brain vasculature in aged animals showed predisposition to increased oxidative stress, activation of NADPH oxidase [26], and of nuclear enzyme poly(ADP ribose) polymerase (PARP) [27]. Ageing is also associated with increased expression of proinflammatory cytokines in vascular endothelial cells from healthy humans [28] which further favours a prooxidative state. Aged brains show increased matrix metalloproteinase- (MMP-) 2 activity and increased MMP-9 expression upon trauma, along with altered BBB repair mechanisms [29].

Molecular studies of BBB impairment in normal ageing explore only superficially the complexity of underling events, usually addressing only few proteins expression and distribution in one experimental paradigm. Results are generated in animal models and convey towards the conclusion that ageing leads to lower tight junction protein expression and a “leaky” BBB status [30–32]. 

Cumulative damage to mitochondria and mitochondrial DNA caused by ROS accounts for the mitochondrial theory of aging. In Figure 1(iii), we show a typical EM image of rat BBB, where mitochondria are clearly observed in both endothelial and glial cells. EM assessment of BBB in aged laboratory animals might offer a clue about mitochondria content and morphology in different BBB cell types, considering the large number of mitochondria in cerebral endothelium [8]. However, to our knowledge, there are no reports exploring mitochondrial alteration in aged brain endothelia so far. 

Age seems to be a BBB frailty-inducing factor, as aged laboratory animals are more prone to brain oedema formation, ischemic injury, neuronal apoptosis following contusion and earlier onset of neuroinflammation than young littermates [33]. In the same manner, BBB dysfunction in old age was shown to be closely related to white matter lesions and lacunar infarctions [34]. 

There are several studies to address BBB permeability in aged animals (reviewed in [35]), in different experimental models, such as reproductive senescent mouse females [36], or senescence-accelerated mice [37]. They all led to the same conclusion that BBB permeability is altered in aged brain. Nevertheless, how and why this impairment occurs is not clear, and data regarding occludin and claudins expression and distribution in aged brain are scarce. 

An overall assessment of BBB integrity can be obtained by immunohistochemistry methods, which show the albumin or immunoglobulin abnormal presence in the brain parenchyma, by elevated CSF albumin to plasma albumin ratio, or by increased perivascular enhancement at brain magnetic resonance imaging (MRI). In human aged brains serum protein immunostaining shows a “leaky” BBB which, interestingly enough, is not associated, at molecular level, with significant changes in endothelial expression of TJ proteins [38], and BBB leakage seems to show a wide individual variation [39]. 

## 3. Oxidative Stress Inducers at BBB Level

Although oxidative stress has been extensively studied in central nervous system different injuries, not enough data is available yet about its triggers and effectors on BBB. To some extent, as a result of vicious circles generated at molecular levels, it is difficult to separate or clearly indicate the cause and the effect of oxidative stress on BBB.

### 3.1. Hypoxia

Hypoxia is probably the best documented pathological process that induces BBB opening. It can be studied *in vitro*, by exposure of cell cultures to a mixture of hypoxic gas (95% N_2_/5% CO_2_; 99% N_2_/1% O_2_) or to pure NO_2_ and *in vivo*, by exposure of animal models to low oxygen air (6–8% O_2_) or ligation of cerebral arteries. Permeability may be further assessed by abnormal transport across BBB of large molecules, such as albumin, labelled dextrans, immunoglobulins, or labelled monocyte migration. Proposed mechanisms for altered permeability include increased exposure to free radicals [40] and/or inflammatory cytokines, such as IL-6 and TNF-*α* [41], activation of MMPs and downregulation of their tissular inhibitors (TIMPs) [42] and induced NOS expression [43], all of them ultimately reflected in the levels of tight junction protein expression.

Opening of BBB in hypoxia/reoxygenation studies is well confirmed in animal models and occurs earlier in aged animals versus young ones [44–46], following a biphasic pattern documented *in vivo* by MRI studies [47, 48].

Hypoxia is known to change BBB permeability and TJ protein expression in cerebral capillaries [49]. Lipid raft-associated occludin oligomeric assemblies were shown to be internalized during hypoxia [50] and ZO-1 and occludin sub-cellular localization correlated with increased paracellular permeability [51]. Reports of claudins expression during ischemia/reperfusion experiments are, however, contradictory. This can be at least partially explained by different experimental paradigms used in different studies.

### 3.2. Inflammation, Proinflammatory Cytokines, and Chemokines

Both normal ageing and neurodegenerative disorders are characterized by a degree of neuroinflammation [52]. In the CNS, proinflammatory cytokines are overexpressed as a result of intense/prolonged oxidative stress and are considered marks of neuroinflammation, a well-proven pathogenic mechanism in Alzheimer's disease (AD) and other neurodegenerative conditions. Cytokines, such as IL-1, IL-6, and TNF-*α*, are increased in plasma and CSF of acute ischemic stroke patients and seem to be associated with increased risk of worsening or recurrence [53–55]. High levels of plasma IL-6, associated with high CRP, seem to be associated with risk of vascular dementia (VaD) [56], and increased levels of IL-6 and TNF-*α* are also associated with senescence and frailty in old age [57]. Along with other cytokines, growth factors and plasma proteins, IL-1*α*, IL-8, and TNF-*α* were proposed as biomarkers able to distinguish AD from controls [58, 59]. A TNF-*α* inhibitor is reported to improve aphasia in demented patients [60, 61]. Proinflammatory cytokines are important regulators of MMPs and TIMPs expression [41]. In particular, TNF-*α*-mediated stimulation of MMP expression and synthesis is considered to be an important link between the proinflammatory cytokine network and the local increase of MMP proteolytic activity [41]. 

Along with TNF-*α*, IFN-*γ* has also been repeatedly reported to modify tight junction barrier function in various polarized epithelia [62–64]. Treatment of cell culture with IFN-*γ* led to decreased protein expression and relocalization of ZO-1 and occludin, occludin and JAM-A [65], in a time and dose-dependent manner. According to Scharl et al., AMP-activated protein kinase (AMPK) in concert with other signals induced by IFN-*γ*, seems to play a role in mediating reduced epithelial barrier function [66]. Chemokines CCL-2 and CXCL-8 are also reported to be responsible for increased BBB permeability, CCL-2 being produced by both astrocytes and endothelial cells in the late phase of hypoxia/reoxygenation-induced BBB disruption [67].

Some of these cytokines and chemokines appear to exclusively affect the paracellular permeability (e.g., IL-1*β* and CXCL8), while some others predominantly act to increase transcellular permeability (e.g., TNF-*α*) [18]. Experimentally induced peripheral inflammation also increases BBB permeability and leads to decreased occludin expression [68] and increased expressions of claudin-3 and 5 [69].

A common experimental animal model used for BBB breakdown in neuroinflammation is the experimental autoimmune encephalomyelitis (EAE), used for the study of multiple sclerosis (MS). An important aspect in the etiopathogeny of MS is loss of immune-privileged environment of the brain and extravasation of leukocytes across the BBB, through chemokine-chemokine receptor interaction. Use of mice with targeted deletions of certain chemokines and their receptors revealed a role for CCL2 and CCR2 in the induction of EAE via effects on infiltrating monocytes [70]. CXCL12 relocation in MS and EAE at the level of the postcapillary venules appears to strongly correlate with the perivascular infiltration of T-cells [71]. CCL19 protein levels in lysates of brain tissue as well as CSF samples were found to be elevated in MS [72]. Regarding the molecular alterations of BBB TJ proteins, in EAE affected mice were noted a coincident loss of both claudin-5 and occludin normal junctional staining patterns [73] and loss of claudin-3 expression that correlated with immune cell infiltration into the CNS and BBB leakiness [74]. Interestingly, although increased expression of claudin-1 in a transgenic EAE mouse model sealed the BBB for paracellular traffic of large molecules, it did not seem to influence immune cell trafficking across the BBB, nor the severity of evolution of the disease [75].

### 3.3. Beta-Amyloid (A*β*) Peptides and Cerebral Amyloid Angiopathy

AD-related BBB disruption is documented in both animal models [76, 77] and human brains [78]. A*β* peptide, one of AD major pathogenic operators, is considered a strong redox active agent capable of generating peroxide in the presence of metals [79]. Soluble A*β* species have been linked to decreased cytochrome C oxidase activity in the Tg2576 mouse model of AD and were shown to enter the mitochondria and cause a signalling amplification that inactivates SOD-2 and generates additional free radicals [80]. A*β* peptides are known to affect brain small blood vessels by inducement of cerebral amyloid angiopathy (CAA), found in 90% of AD patients and 50% of 90-year-old population [81]. A*β*-loaded capillaries, surrounded by NADPH oxidase-2 (NOX-2)-positive activated microglia are characterized by a dramatic loss of occludin, claudin-5, and ZO-1. Importantly, same brain sections showed abundant vascular expression of the A*β* transporter receptor for advanced glycation end-products (RAGE) [82], that was recently demonstrated to function as a signal transducing cell surface receptor for A*β*1-42, to induce ROS generation from NADPH oxidase [83]. A*β*1-40 perivascular deposition was reported to decrease expression of TJ proteins claudin-1 and claudin-5 and to increase expression of MMP-2 and MMP-9, in both AD brain microvessels and brains of AD transgenic mice [78]. In the neocortex and hippocampus of aged Tg2576 mice, the ratio of occludin to *β*-actin was reduced by nearly half, when compared to age-matched wild type controls, but also with young transgenic mice [84].

*In vitro*, in cellular barrier models, A*β* treatment increases endothelial permeability, effect documented for both A*β*1-40 [85] and A*β*1-42 [86], while tight junction protein expression is controversial (Table 1). In cultured endothelial cells, A*β*1-42 induced enhanced permeability by disruption of ZO-1 expression in the plasma membrane and increased intracellular calcium and matrix metalloproteinase (MMP) secretion. Neutralizing antibodies against RAGE and inhibitors of calcineurin and MMPs prevented A*β*_1-42_-induced changes in ZO-1, suggesting that A*β*-RAGE interactions alter TJ proteins through the Ca^2+^-calcineurin pathway. Consistent with these *in vitro* findings, Kook et al. found disrupted microvessels near A*β* plaque-deposition areas, elevated RAGE expression, and enhanced MMP secretion in microvessels of AD mouse brains [86].

### 3.4. Excessive Alcohol Consumption

Excessive alcohol consumption is a known etiologic factor for cognitive impairment and dementia in humans and long-term treatment of adult laboratory rats with 20% ethanol in drinking water ad libitum resulted in cognitive decline, cholinergic dysfunction, and BBB leakage [87]. Ethanol (EtOH) effects are at least partially mediated by ROS, since, in these mice, superoxide production under basal conditions and in the presence of ADP and NAD(P)H, was increased [88]. In laboratory rats, EtOH consumption has previously been reported to associate increased oxidative stress and cytochrome P450-2E1 activation [89]. EtOH-induced activation of MMP-3/9 led to subsequent degradation of BBB proteins, occludin, claudin-5, and ZO-1 [90].

*In vitro*, EtOH induces ROS generation and ROS-nitrated protein accumulation in BMVEC [91]. At BBB level, EtOH or its metabolite acetaldehyde increases leakage and TJ protein phosphorylation [92]. Similar effects have been reported in other barriers, such as blood-air barrier [93], or other types of TJ-dependent polarized epithelia [94, 95].

## 4. Mediators of Oxidative Stress and Their Effects on Tight Junction Proteins

### 4.1. Reactive Oxygen Species

ROS are the main operators of oxidative stress and are responsible for altering protein structure, DNA denaturation, and lipid peroxidation and may act as messengers in redox-signalling systems [49]. In addition to causing cellular oxidative damage to biomolecules, hydroxyl radicals can also react with A*β*, triggering the formation of dityrosine cross-linking between A*β* peptides which leads to enhanced oligomerization and aggregation [96]. In oxidative-inducing conditions, a number of mechanisms have been proposed to trigger ROS generation, with enzymes such as xanthine oxidase, cyclooxygenase, leukocyte NADPH oxidase, and uncoupled endothelial NOS (eNOS) and mitochondria as putative sources [97]. Increased oxidative stress associated with aging further worsens the outcome of a stroke and favours onset of dementia. Common ROS that are deleterious for the vascular endothelium as well are superoxide, hydroxyl radical and hydrogen peroxide, found in concentrations depending of the balance between oxidases, such as NADPH oxidases (Nox enzymes) and superoxide dismutases (SOD). The impact of ROS on BBB function has been documented on SOD deficient mice, in which ischemia/reperfusion experiments demonstrated increased endothelium permeability to large molecules [98]. The hydrogen peroxide is more stable than superoxide, diffuses easily across cell membrane, can stimulate NADPH oxidase in vascular cells and thus further increase levels of superoxide [99]. 

In *in vitro* models, superoxide and other ROS increase permeability of the BBB in a time- and concentration-dependent manner [100, 101]. The reports on TJ proteins expression yielded contrasting results; however, BBB functionality was altered regardless of experimental paradigm. For instance, Lee et al. reported ROS-induced BBB impairment, quantified by transepithelial electrical resistance (TER) measurements, associated with a slight but significant increase in occludin expression [102], whereas Schreibelt et al. provided evidence that short-term oxidative stress-induced redistribution of occludin and claudin-5, with Western blot evidence of loss of these proteins expression [103].

### 4.2. Nitric Oxide

NO is a signalling molecule and a potent vasodilator, generated at the BBB level by eNOS, from L-arginine, a process that requires 5,6,7,8-tetrahydro-l-biopterin (BH4) as coenzyme. Apart from the constitutive isoform of NOS, endothelial cells also produce inducible NOS (iNOS), activated by interleukins and TNF-*α* [104]. Activation of iNOS is long-lasting and leads to an increased production of NO, as compared to constitutive isoform. Transgenic iNOS knockout mice develop brain pathology characteristic of AD (amyloid plaques, tau phosphorylation, and neuronal loss) indicating the NO has a protective role [105]. The peroxinitrite resulted from NO during oxidative stress has neurotoxic effects, similar to other ROS, via lipid peroxidation and DNA damage [106], and its presence is documented in astrocytes, neurons as well as blood vessels of AD brains, both in humans and mouse models of AD [80]. Generation of peroxynitrite from NO and superoxide takes place at a faster rate than the dismutation of superoxide by SOD enzymes and results in the loss of normal NO-mediated signalling. Thus, the local concentration of superoxide is a key determinant of the biological half-life of NO [98].

Interestingly, in certain conditions such as reduced levels of BH4, eNOS itself can produce superoxide, a process referred to as “eNOS uncoupling,” in which oxygen becomes terminal electron acceptor instead of L-arginine [107]. NO does not influence the function of BBB during normoxia, but seems to confer protection during ischemia [108].

### 4.3. Lipid Peroxidation Products

Lipid peroxidation usually designates the oxidative damage of polyunsaturated fatty acids by free radical chain reactions when exposed to O_2_ in the presence of trace metal ions. Studies of chain reactions in purified chemical systems show that a single initiation event can oxidatively damage 200 to 400 lipid molecules before two radicals react to eliminate the unpaired electrons and terminate the reaction sequence [109]. Lipid peroxidation causes damage at several levels by generation of various reactive aldehydes, such as 4-hydroxynonenal (4-HNE), that can alter the phospholipid asymmetry of the membrane lipid bilayer, and other products of lipid peroxidation, that can react with mitochondrial enzymes and cause disruption of mitochondrial energetics, increase of free radicals release and further oxidative stress [80]. A*β* peptides exert their oxidative effect on membrane lipids as well and there is a strong correlation between lipid peroxides, antioxidant enzymes, amyloid plaques, and neurofibrillary tangles (NFTs) in AD brains [3]. The composition of brain in phospholipids is unique; therefore, specific intermediates are produced upon lipid peroxidation [110]. These intermediates may diffuse into the blood stream and affect red blood cell membrane, as proven by Skoumalova et al. [111]. Oxidized low-density lipoprotein (ox-LDL), which is a hallmark feature of atherosclerosis acts as a stress signal and plays an influential role in BBB permeability [112]. The mechanisms by which lipid peroxidation affects BBB are not elucidated yet, but it has been proven that 4-HNE increases permeability of an *in vitro* barrier model [113]. In hyperlipemic laboratory mice, lipid peroxidation activates MMP-2/9, which in turn induces RhoA activation, a small GTPase known to phosphorylate TJ proteins and further destabilise BBB [114].

### 4.4. Matrix Metalloproteinases

Produced by activated microglia, MMPs are responsible for breaking down of endothelial basal lamina of BBB [57]. The main MMPs studied in relation to BBB alteration are MMP-2 and MMP-9, the first being constitutively expressed in CNS and the latter a marker of neuroinflammation [115]. Expression of MMP-9 within 24 h of an ischemic insult has cellular specificity, being primarily confined to the brain endothelium [116]. As already stated above, MMPs activity is balanced by their endogenous inhibitors, the TIMPs. Direct intracerebral injection of MMP-2 results in opening of the BBB with subsequent haemorrhage, effect that can be prevented by co-administration of TIMP-2 [117]. Blocking MMP-2 activation using either a selective inhibitor or a neutralizing antibody demonstrated that this enzyme is responsible for ischemia-induced occludin degradation. Interestingly, claudin-5 seems to be downregulated by different mechanisms, involving caveolin-1 [118]. On the other hand, Bauer et al. argued that hypoxia-induced oedema formation is mediated by MMP-9-dependent TJ rearrangement by a signalling cascade involving trophic factors, such as VEGF [119].

## 5. Signalling Pathways Affecting Tight Junction Proteins Phosphorylation Status in Oxidative Environments

Several reports of Saitou et al. showed in different experimental models that absence of occludin expression does not disrupt organization and function of TJs [120–122]. Therefore, TJ proteins emerged as possible signalling molecules. Indeed, there are several phosphorylation sites in the C-terminus sequence of occludin and claudins and phosphate addition in these domains increase protein internalization [65]. As a result, phosphorylation promotes an increase in BBB “leakiness.” These phosphorylation sites are found within consensus sequences for protein kinase C (PKC) and protein kinase A (PKA) [123] and it was further proven that some PKC isoforms are involved in occludin, claudins, and ZO species phosphorylation in normal and hypoxic conditions. Hypoxia-induced BBB changes involved increased paracellular permeability via a PKC activity-dependent mechanism, in both *in vitro* and *in vivo* conditions [124].

Cytoplasmic relocation of occludin, claudin-1, and ZO-1 were documented in Ras-transformed Madin-Darby canine kidney epithelial cells (MDCK), effect that was specifically reversed by mitogen-activated protein kinase 1 (MEK-1) inhibition [125]. As demonstrated by Wang et al., an occludin mutant lacking the first extracellular loop rescued cells from Raf-1-mediated transformation [126]. Furthermore, different small GTPases, such as Raf-1 [127], Rho, Rac [128], were shown to influence the expression of occludin and claudin-1 in different epithelial models. Moreover, addition of ROS in cell culture media of immortalized rat endothelial brain cells significantly induced transient PKB phosphorylation and subsequent activation, through RhoA activation [103]. Occludin undergoes phosphorylation at Tyr residues during the disruption of TJs by oxidative stress and acetaldehyde [129]. Occludin and claudin-1 protein expression seems to be influenced by Glycogen Synthase Kinase-3 *β* (GSK-3*β*) as well, inhibition of this kinase leading to decreased TJ protein levels [130].

## 6. Conclusions

Oxidative stress has been involved for a long time and by overwhelming scientific data as a main pathogenic event in brain ageing and neurodegeneration. BBB, as crucial gate of brain-blood molecular exchange, seems to be affected by oxidative stress inducers in early stages of different brain diseases. Further studies are needed to understand which is the relationship between ROS deleterious effects on endothelial cells, BBB impairment, and progress of neurodegeneration, and how specific BBB drug targets can be approached in the future.

## Figures and Tables

**Table 1:** Expression of tight junction proteins in various cellular models of oxidative stress.

BBB *in vitro* model	Type of experiment	Special conditions	Documentation of BBB permeability increase	Tight junction proteins alterations	Reference
BBMEC monolayers	Hypoxic stress	Glial conditioned-media treatment	Permeability studies with [14]-sucrose	Claudin-1 shows a significant increase following hypoxic stress	[21]

BBMEC monolayers	Hypoxia/reoxygenation	none	TEER measurements and [14]-sucrose transfer across the barrier	Significant increase in expression of occludin, ZO-1, and ZO-2	[131]

Rat GP8/3.9 cells	ROS generating environment by a mixture of xanthine oxidase and hypoxanthine	—	TEER FITC-dextran permeability across the barrier	Decrease of occludin and claudin-5 expression after exposure to oxidative environment	[103]

PBMEC	Hypoxia	Coculture with astrocytes/C6 glioma cells	TEER and passage of [3H]inulin	Decreased ZO-1 immunoreactivity at regions of cell-cell contact	[43]

BMVECs on a 8.0 *μ*m matrigel-based insert	MMPs aggression	Coculture with leukemic cells	40 kDa dextran-FITC flux by flow cytometry analysis	Downregulation of ZO-1, claudin-5, and occludin	[132]

hCMEC/D3 (immortalized human BEC line)	A*β* peptides treatments	—	permeability to the paracellular tracer 70 kD FITC-dextran	Decrease in the occludin level, whereas claudin-5 and ZO-1 were unaffected	[85]

Human BMVEC	Exposure to ROS	—	TEER and monocytes migration studies	Decreased occludin and ZO-1 total content, whereas claudin-5 expression depended on the type of stressor used	[91]

BBMEC: bovine brain microvessel endothelial cells.

TEER: transendothelial electrical resistance.

PBMEC: primary cultures of porcine brain-derived microvascular endothelial cells.

BMVEC: brain microvascular endothelial cells.

ROS: reactive oxygen species.

MMPs: matrix metalloproteinases.
